# Uncommon Presentation: A Case Report on a Rare Vulvar Fibroadenoma

**DOI:** 10.7759/cureus.53834

**Published:** 2024-02-08

**Authors:** Rida Noor, Aman Kumar, Jaweria Pervaiz, Ramsha Ali, Fnu Sanjna

**Affiliations:** 1 Pathology, Faisalabad Medical University, Faisalabad, PAK; 2 Medicine, Shaheed Mohtarma Benazir Bhutto Medical University, Larkana, PAK; 3 Histopathology, Chughtai Institute of Pathology, Lahore, PAK; 4 Pediatrics, Dow University of Health Sciences, Karachi, PAK; 5 Medicine, Shaheed Mohtarma Benazir Bhutto Medical University, Karachi, PAK

**Keywords:** benign, vulva, glandular and epithelial neoplasms, ectopic fibroadenoma, neoplasm

## Abstract

Vulval fibroadenoma is an uncommon, benign tumor that originates from ectopic breast tissue or mammary-like glands in the anogenital region. Only a limited number of cases have been documented in medical literature. Typically occurring in young and middle-aged women, this condition, when surgically removed, generally exhibits a favorable prognosis with a low recurrence rate. We report a case of vulvar fibroadenoma wherein the patient exhibited a groin region mass. The mass was then excised and examined histologically. Histological examination of the polypoidal tissue section unveiled a clearly defined lesion comprising both epithelial and stromal components.

## Introduction

Fibroadenomas are common benign growths occurring in the female breast. They can be easily removed through surgical methods. When observed closely, they present as solid, well-defined, oval nodules with a smooth, bosselated outer surface and a tan-gray, bulging, lobulated inner surface, often displaying visible slit-like spaces. However, their gross appearance can range from soft and mucoid to highly fibrotic and calcified. Moreover, mammography can detect non-palpable fibroadenomas, revealing them as masses, microcalcifications, or a combination of both. Vulval fibroadenomas can be identified through fine-needle aspiration cytology (FNAC) and a histopathological analysis of the tissue. Ectopic breast tissue is an exceptionally uncommon occurrence, with the axilla being the most prevalent location and the vulva ranking as the second most infrequent site [1]. It is estimated that ectopic breast tissue affects 2% to 6% of women in the general population, with the vulva being recognized as an unusual location for this condition [2]. Ectopic tissue in women is most often located in the axilla but can also be found anywhere along the milk line or even beyond it [3].

## Case presentation

A 42-year-old female patient visited the outpatient clinic with a painless, well-defined mass in the vulva, and it has been growing gradually in size in the past few years. During the initial clinical examination, a vulval lipoma was identified as it was a painless, solitary, mobile, and subcutaneous mass not linked with epidermal changes. The patient underwent surgery because of pressure symptoms, and the mass was excised during the procedure. On gross examination, the specimen was received in formalin consisting of skin-covered polypoidal tissue measuring 7.5x7.5x3 cm. The overlying skin ellipse was unremarkable. A histological examination revealed a well-circumscribed lesion composed of both epithelial and stromal components. Ducts are lined by the inner ductal epithelial and outer myoepithelial layers. The ductal lining cells are uniform, and their lumina are compressed to form slit-like structures. The surrounding stroma shows spindle cells arranged in fascicles with elongated nuclei and a moderate amount of cytoplasm. No atypia, stromal overgrowth, or evidence of malignancy is seen. The patient underwent surgery, and she is now in a normal state of health.

**Figure 1:**
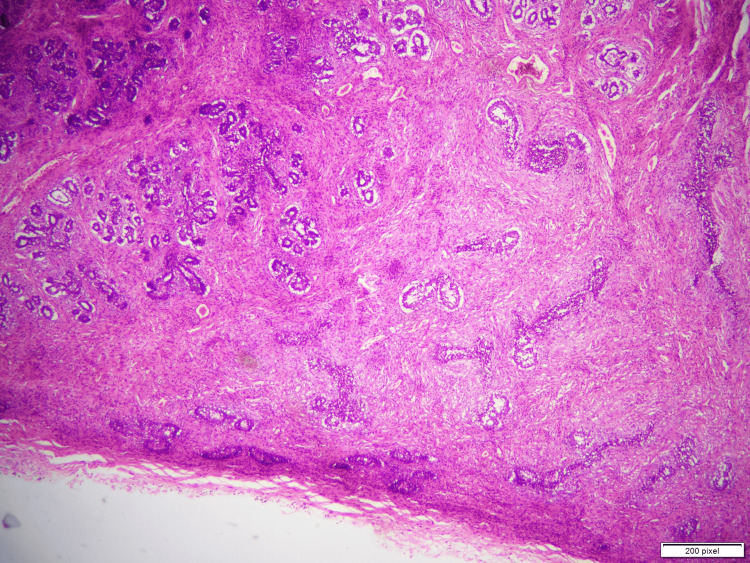
Low-power image depicts a well-circumscribed lesion with preserved lobular architecture.

**Figure 2:**
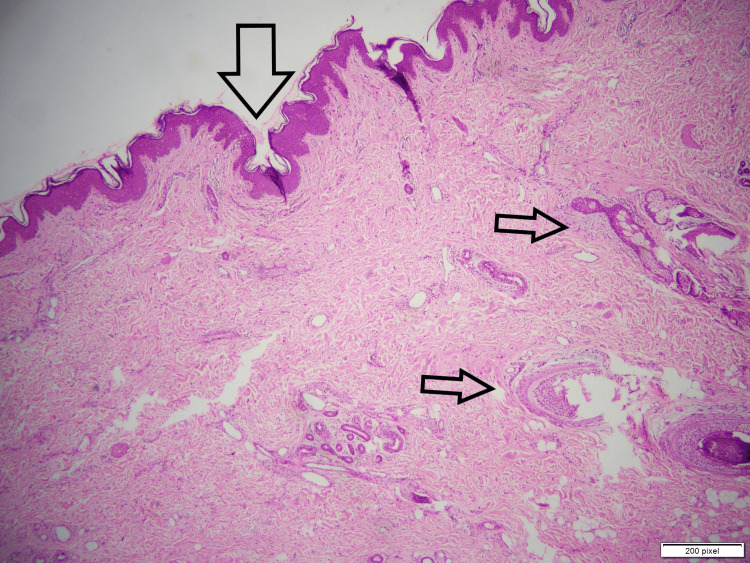
Skin tissue with underlying dilated ducts. Large arrow: epidermis. Small arrows: dilated ducts filled with secretions.

**Figure 3:**
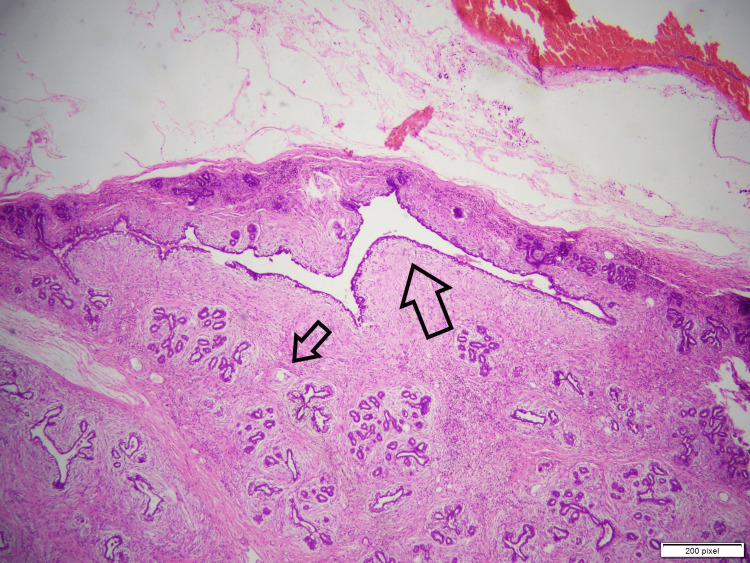
Well-circumscribed neoplasm comprised of intact lobular architecture. Large arrow: glandular proliferation (pericanalicular growth pattern; glands retain open lumen). Small arrow: the stromal component.

**Figure 4:**
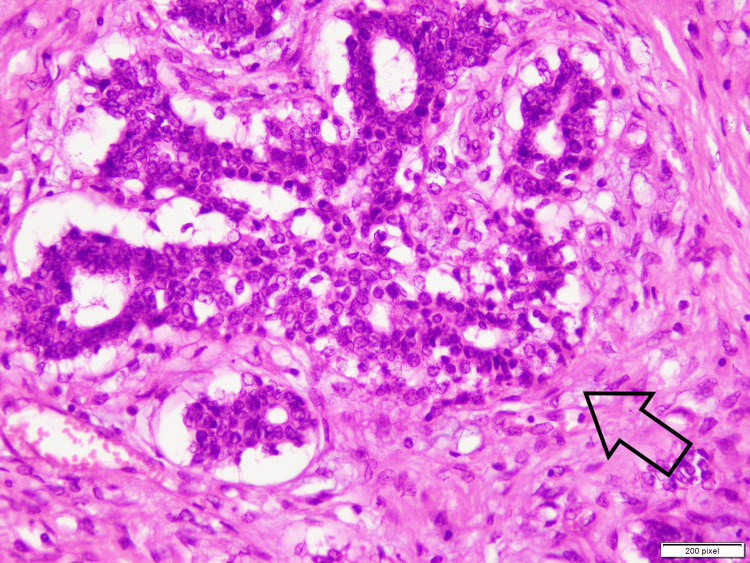
High-power image shows both glandular and stromal proliferation. The arrow shows that glands are lined by two-cell population: ductal and myoepithelial cells.

Figure 1 shows skin tissue with underlying dilated ducts beneath. Figure 2 depicts a well-circumscribed neoplasm that displays an intact lobular structure. Figures 3-4 display that under higher magnification, both glandular and stromal proliferation are evident. The glands are characterized by the presence of a two-cell population: ductal and myoepithelial cells.

## Discussion

While rarely, ectopic breast tissue can be found in the vulva. This could be attributed to the vulva’s position at the lower end of the primitive mammary ridge. These lesions can exhibit both benign and malignant transformations as they often react to hormones similarly to those in the normally located breast [4].

The tumor’s behavior closely resembles that of breast tumors. Typically, excision results in a favorable prognosis, with rare occurrences of recurrence. The presence of ectopic breast tissue in the vulva was first documented in 1872 [5]. Ectopic breast tissue or tissue resembling mammary glands in the anogenital region demonstrates the presence of hormone receptors that can be identified through immunohistochemistry. This tissue can develop both benign and malignant lesions, resembling those found in normal mammary tissue, and may even exhibit lactational changes. The contribution of elevated serum estradiol relative to progesterone in the development of conditions like breast fibroadenoma warrants additional investigation [5,6]. Most cases documented in medical literature typically pertain to individuals either during pregnancy or shortly after giving birth. Our case underscores the potential for ectopic breast tissue to manifest beyond the intrapartum or postpartum phases. While the exact source of ectopic breast tissue remains a subject of debate, the presence of benign breast tissue in the vicinity of these lesions lends support to the notion that these originate from ectopic breast tissue rather than anogenital mammary-like glands [7]. The ducts exhibit positivity for ER, PR, SMA, S100, CK, and EMA. These tumors are benign and share behavior like those observed in the breast. Generally, excision leads to a favorable prognosis, and recurrence is observed only in 3% of cases [8].

## Conclusions

To summarize, we present a case of a rare condition called vulvar fibroadenoma. The mass was successfully removed, and the patient experienced a positive outcome, indicating the benign nature of the lesion. The infrequency of vulvar ectopic breast tissue can pose a diagnostic dilemma for both healthcare providers and anatomical pathologists. We believe that this case report will enhance clinicians’ awareness of vulvar ectopic breast lesions. After performing an excisional biopsy and obtaining histological confirmation of the lesion, it is possible to provide reassurance to the patient. While fibroadenomas in the vulva are uncommon occurrences, careful consideration should be given to them in the differential diagnosis, especially when examining the cytomorphological features of fine needle aspiration (FNA) biopsy of the mass.
